# Physiotherapy for poliomyelitis: a descriptive study in the Republic of Congo

**DOI:** 10.1186/1756-0500-7-755

**Published:** 2014-10-23

**Authors:** Silvia Mancini, Matthew E Coldiron, Sarala Nicholas, Augusto E Llosa, Isabelle Mouniaman-Nara, Joseph Ngala, Rebecca F Grais, Klaudia Porten

**Affiliations:** Epicentre, Paris, France; Médecins Sans Frontières, Rome, Italy; Médecins Sans Frontières, Paris, France; Ministry of Health, Pointe Noire, Republic of the Congo

**Keywords:** Poliomyelitis, Physiotherapy, Infectious disease, Paralysis

## Abstract

**Background:**

A large poliomyelitis outbreak occurred in 2010 in the Republic of Congo. This paper describes the demographic and clinical characteristics of poliomyelitis cases and their outcomes following physiotherapy.

**Findings:**

Demographic and clinical data were collected on 126 individuals between November 23, 2010 and March 23, 2011. The male/female ratio was 2.5 and the median age was 19 years (IQR: 13.5-23). The most severe forms of the disease were more common in older patients, 81 of the 126 patients (64.3%) had multiple evaluations of muscle strength. Among patients with multiple evaluations, 38.1% had improved strength at final evaluation, 48.3% were stable and 13.6% had decreased strength.

**Conclusions:**

Most acute poliomyelitis patients receiving physiotherapy had improved or stable muscle strength at their final evaluation. These descriptive results highlight the need for further research into the potential benefits of physiotherapy in polio affected patients.

## Background

After 10 years with no detected wild poliovirus transmission in the Republic of Congo [1], a large outbreak of poliomyelitis occurred in 2010. The outbreak started with an unusual accumulation of cases of acute flaccid paralysis (AFP) in September and October 2010 in Pointe Noire, the second largest city in the country and the main commercial centre.

The World Health Organization (WHO) defines AFP as a syndrome characterized by rapid onset of weakness of an individual’s extremities, often including weakness of the muscles of respiration and swallowing, progressing to maximum severity within 1–10 days. The term ‘flaccid’ indicates the absence of spasticity or other signs of disordered central nervous system (CNS) motor tracts such as hyperreflexia, clonus, or extensor plantar responses [2]. AFP is the most common sign of acute polio, and is used for poliomyelitis surveillance and often during poliomyelitis outbreaks.

During the Pointe Noire outbreak, Poliovirus type 1 was identified in specimens tested at a WHO-accredited laboratory. AFP cases were classified as laboratory confirmed polio, clinical polio or non-polio cases by the National Polio Expert Committee (NPEC). An AFP case was considered as clinically confirmed polio if it occurred in a person with a strong likelihood of having poliomyelitis based on clinical presentation, in a person with residual paralysis at least 60 days after onset and in a person who lived in a province which had at least 1 confirmed laboratory case of polio.

Overall in Pointe Noire, 378 cases were clinically confirmed, 64 cases were laboratory confirmed and 3 were clinically compatible. The outbreak affected mainly the adult population (57.4% of cases were 15–24 years of age) with a large number of severe cases and a high case fatality rate, especially in the male population. Further details of this epidemic have been described elsewhere [3–5].

At the request of the Ministry of Health, Médecins Sans Frontières (MSF) provided medical services and physiotherapy for AFP patients in the two public hospitals of Pointe Noire, Adolphe Cissé and Tié-Tié between November 23, 2010 and March 23, 2011. The interventions were provided in these two public hospitals because all AFP cases were referred there.

Although recommended by the WHO [6], the effects of physiotherapy on muscle strength in the acute poliomyelitis phase remains poorly documented and the bibliography available on that subject is outdated. A few studies have addressed the potential role of physiotherapy on the course of polio disease and muscle strength and functioning [7–9], showing that physiotherapy is an effective treatment of polio-related problems and can improve muscle function. A study carried out on 70 polio cases treated in 1952–1953 showed improvements in muscle strength after physiotherapy, for some patients, as long as 6 months after symptom onset [10]. In another study on poliomyelitis, the phase of potential recovery of muscle strength varied from a few weeks to a maximum of 2 years [11]. Targeted physiotherapy at different stages of the illness may therefore help to restore strength, to prevent deformity, and to rehabilitate patients.

Here, we describe the demographic and clinical characteristics as well as the physiotherapeutic outcomes of patients admitted to Adolphe Cissé and Tié Tié Hospitals during one of the largest poliomyelitis epidemics in the eradication era. Due to limited resources and logistical infrastructure in the Republic of Congo, the description focuses on manual measurements of muscle strength.

## Methods and analysis of data

We performed a retrospective analysis of program data to describe patients’ responses after physiotherapy. Using the WHO case definition, suspected poliomyelitis during the epidemic was defined as "any case of acute-onset flaccid paralysis (AFP), including Guillain-Barré syndrome, in a person under 15 years of age for any reason other than severe trauma, or paralytic illness in a person of any age in which polio is suspected" [12]. Here we describe the laboratory-confirmed and clinically-compatible polio cases as classified by the NPEC who were inpatients at the two hospitals between November 23, 2010 and March 23, 2011, regardless of the date of diagnosis.

The medical and physiotherapeutic interventions included both an inpatient and outpatient component. A protocol on physiotherapy sessions and measurements was defined at MSF OCP headquarters in Paris. It was applied systematically in the field by four expatriate physiotherapists along with 6 local health professionals trained and supervised by the experienced physiotherapists. During the physiotherapy program, muscle strength was measured using the Medical Research Council scale of 1943 (Table 1).

**Table 1:** **Medical research council grading of muscle strength**

Grade 0	No movement is observed
Grade 1	Only a trace or flicker of movement is seen or felt in the muscle, or fasciculation is observed
Grade 2	Movement is possible only if the resistance of gravity is removed
Grade 3	Movement against gravity is possible but not against resistance of the examiner
Grade 4	Muscle strength is reduced but muscle contraction can move joint against gravity and resistance
Grade 5	Muscle contracts normally against full resistance

At admission to hospital, life-saving measures were prioritized. The treatment of patients was organized according to different type of paralysis. Patients were treated with vitamin B and antibiotics when indicated, and an adequate caloric intake was assured. Prevention of pressure sores and pain control were provided together with circulatory massages. Cases presenting weakness of respiratory and throat muscles were treated with mechanical ventilation. In cases with dysphagia, nutritional intake was given through a naso-gastric tube. Deformities were prevented by passive movement in short sessions several times a day, and by providing supportive equipment.

After discharge from hospital, twice-weekly outpatient sessions focused on patient independence in daily activities. Different techniques were used to strengthen muscles, and breathing exercises were carried out to prevent or reduce infections and to maintain sufficient vital capacity. To prevent muscle contractures, paralyzed limbs were passively mobilized; active assisted mobilization and strength training for weak and recovering muscles was also provided. To keep the limb in the corrected position between sessions, braces were used. Patients were trained in walking with supportive equipment.

The treatment of residual paralysis and muscles measuring <3 on the MRC scale consisted of increasing resistance in the weakest muscles and coordination of muscle groups. Moderately weakened muscles (muscles measuring ≥3 on the MRC scale) were exercised with the goal of increasing strength. Range of motion of major joints was regularly assessed. Patients were evaluated for orthoses and other durable medical equipment. Individual psychotherapeutic sessions were also organized to provide support in coping with the trauma of sudden paralysis and with social stigma.

During the outpatient period, the degree of paralysis was recorded at each visit. Muscle strength was evaluated using the MRC Scale at baseline and once a month. A patient was classified as having severe disease if he or she had an MRC scale between 0 and 2 in more than 2 limbs. Treatment outcome was defined as change in MRC score between the first and the last visit, where improvement was an increase of muscle strength by at least 1 point, worsening was a decrease in muscle strength by at least 1 point and stable where no change was observed.

Patients were divided into 4 age groups. Patients aged less than 5 years (the age group traditionally affected by poliomyelitis), patients aged 6 to 15 years (school aged children), patients aged 16 to 24 years (young adults) and patients aged older than 25 years (adults).

Data were collected by MSF clinicians at the time of consultation. Three types of standardized data forms were completed: demographic, clinical and physiotherapy data. Data were entered in EpiData version 3.1 (Odense, Denmark) and analyzed using Stata version 11 (College station, Texas, USA).

Differences in patient characteristics (age, sex, delay in initiation of physiotherapy, and individual limb strength) were compared using chi-squared tests in those patients receiving only one muscle strength evaluation and those with multiple evaluations. The Fishers Exact test was performed to assess differences between patient age groups. Medians were compared using the Wilcoxon test.

### Ethical considerations

This analysis used routine monitoring data collected by MSF-OCP in collaboration with the Ministry of Health of the Republic of Congo as described in a memorandum of understanding. This is a normal operating procedure for medical interventions during public health emergencies. Authorization from the Ministry of Health of the Republic of Congo was obtained prior to data collection. Privacy and confidentiality of patients were ensured. All data were anonymized when entered into the database and identification numbers were coded. No ethnic or identifying information was encoded. This retrospective description of program data is exempt from review by the MSF Ethical Review Board.

## Findings

A total of 142 patients were diagnosed with poliomyelitis in Adolphe Cissé and Tié-Tié hospitals from November 23, 2010 to March 23, 2011. Among these patients, 13% (19 out of 142) were laboratory confirmed cases, and the rest were designated as probable polio cases by the NPEC. Of these, 5 were lost to follow-up, 1 refused physiotherapy during follow-up, 2 died before hospital discharge and 8 patient files were incomplete. Baseline demographic and medical characteristics of the remaining 126 individuals were analyzed (Table 2). The male to female ratio was 2.5, and the median age of the patients was 19 years old (IQR: 13.5 - 23). The most common presenting symptoms included fever, headache, body pain and joint stiffness with 56 patients (43%) presenting with these symptoms at disease onset. On average, paralysis began 2 days after onset of febrile illness. The most clinically severe forms, such as those involving paralysis of abdominal muscles, were more common in older age groups.

**Table 2:** **Patient characteristics by age group at baseline (N = 126)**

Age group, years ^*^	n (% of total)	Affected limbs, median	Severely affected limbs, median	Abdominal paralysis, n (% of group)	Dorsal paralysis, n (% of group)
0–4	15 (12)	4	1	7 (47)	6 (40)
5–14	20 (16)	4	1	14 (70)	14 (70)
15–24	62 (50)	3	1	40 (65)	39 (63)
≥25	28 (22)	2	1	16 (57)	16 (57)

^*^Age group missing for one patient.

### Physiotherapy characteristics

Among the 126 patients who received physiotherapy, 45 (35.7%) had a single outpatient visit with muscle strength evaluation and were then lost to follow-up. 81 patients (64.3%) had two or more visits with muscle strength evaluation, for a total of 264 visits. Comparing patients with only one muscle strength evaluation to those with more than one, there were no significant differences in age (mean age: 19 years vs. 18 years), gender (32.4% vs. 44.6% females, p = 0.4), or delay in the initiation of physiotherapy (mean delay 54 days vs 54.4 days). There were statistically significant differences between the two groups in terms of MRC scores and the number of limbs affected (Table 3).

**Table 3:** **Severity of weakness in affected limbs in patients with one muscular evaluation (N = 45) and patients with ≥2 muscular evaluations (N = 81)**

Limb	1 muscle strength evaluation, n (%)	≥2 muscle strength evaluations, n (%)	p-value (Pearson)
Left Arm*			
MRC score <3	8 (18)	36 (67)	0.003
MRC score ≥3	36 (82)	45 (33)	
**Right Arm**			
MRC score <3	6 (13)	36 (44)	0.001
MRC score ≥3	39 (87)	45 (55)	
**Left Leg**			
MRC score <3	18 (40)	51(63)	0.013
MRC score ≥3	27 (60)	30 (37)	
**Right Leg**			
MRC score <3	14(31)	57 (70)	0.001
MRC score ≥3	31 (69)	24 (30)	

*One missing value.

Among 81 patients with multiple muscle strength evaluations, 51 (63%) had residual weakness in all 4 limbs, with a median of three severely affected limbs (Table 4). Among patients with dorsal paralysis, the male/female ratio was 2.5 (p = 0.043).

**Table 4:** **Evolution of muscle strength in patients with ≥2 evaluations (N = 81)**

	At first visit		At final visit		
Limbs affected	Number of patients (% of total)	Limbs severely affected, median [IQR]	Limbs severely affected, median [IQR]	Limbs with improved strength, median [IQR]	Limbs with decreased strength, median [IQR]
1	5 (6)	1 [0–1]	1 [1–1]	1 [0–1]	0 [0–2]
2	16 (20)	1 [1–2]	1 [0–1]	1 [0–1]	0 [0–0]
3	9 (11)	1 [0–2]	0 [0–1]	2 [2-3]	0 [0–1]
4	51 (63)	3 [2–4]	3 [2–4]	2 [1–3]	1 [0–1]
Total	81 (100)	2 [1–4]	2 [1–3]	1 [1–2]	0 [0–1]

**Figure 1:**
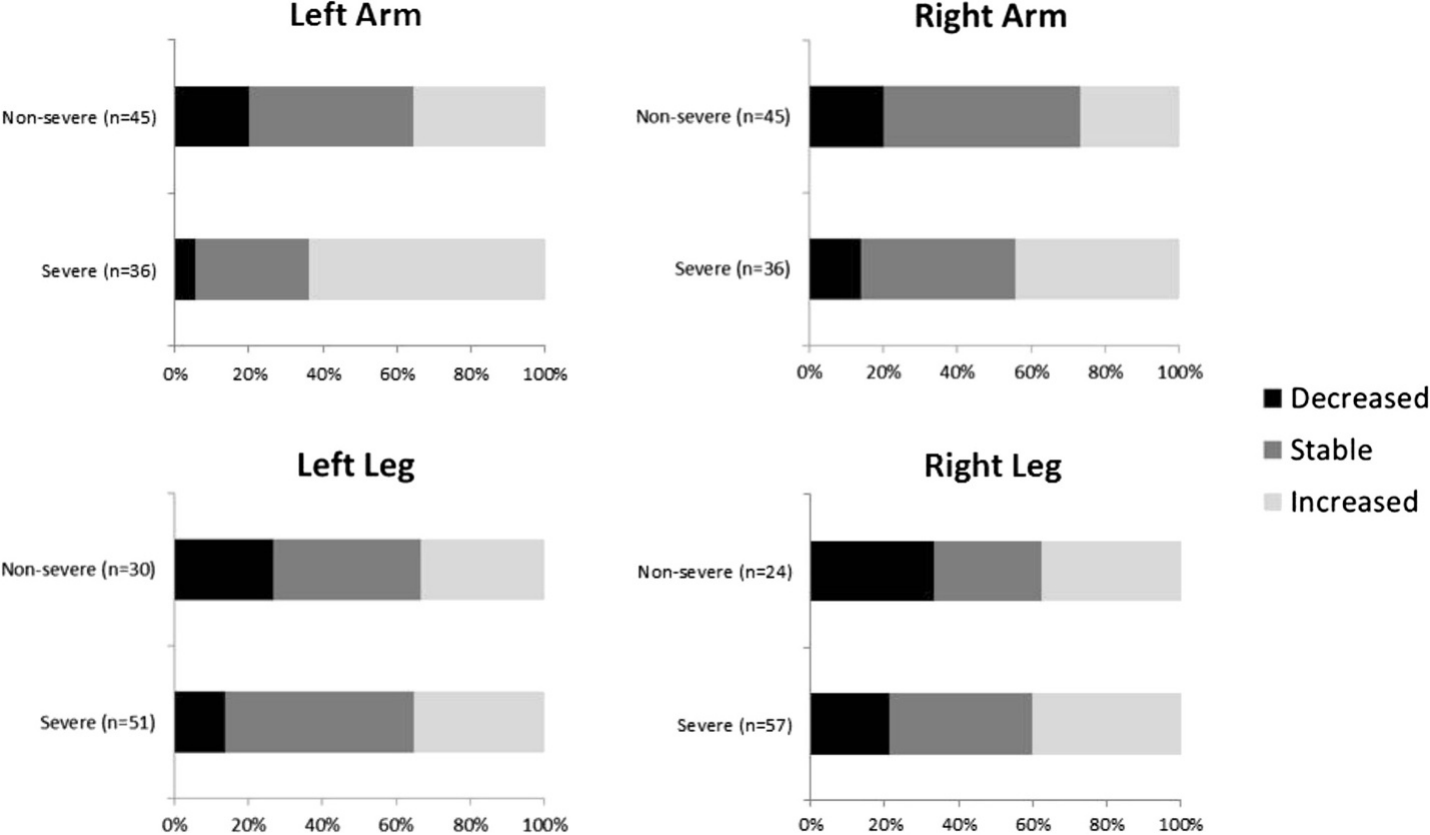
Change in muscle strength between baseline and final visit by severity at baseline (N = 81)*. Change in muscle strength between baseline and final visit is shown for each limb. In all four limbs, patients with severe weakness at baseline are more likely to have improved or stable strength at the final visit. *Severity is defined as MRC score of <3 in a given limb.

Among those with multiple evaluations of muscle strength (Table 4), strength improved in 31 patients (38%), remained stable in 39 patients (48%) and deteriorated in 11 patients (14%). The magnitude of improvement varied from a minimum of 1 point to a maximum of 3 points. Patients who improved or remained stable (86%) were among the most severe patients at baseline. The change in MRC score for each limb is shown in Figure 1.

## Discussion

We have described the demographic and clinical characteristics of a subset of polio patients during a major outbreak in Pointe Noire, Congo, and further describe the potential impact of physiotherapy added to the standard outbreak response.

The results presented here suggest that physiotherapy, administered at each stage of the disease and tailored to meet individual patient needs, could be an important factor for the potential recovery of muscular strength. At the least, we note that only a small minority (14%) had increasing muscle weakness at the end of their physiotherapy. However it is important to note that the outpatient follow-up physiotherapy offered in this setting was an optional adjunct for patients.

These descriptive results are supported by positive results from several recent studies on the utilization of physiotherapy in the post-polio syndrome [13–15]. Post-polio syndrome is a condition which affects polio survivors years after recovery from an initial attack of polio virus and is characterized by slowly progressive muscles weakness, fatigue, and gradual atrophy [16]. Increased understanding of best treatments for post-polio syndrome patients has led to an increased awareness of the potential benefits of exercise and physiotherapy during acute poliomyelitis. Individualised treatment plans (both for individuals and for individual muscle groups) are likely important in these complex patients [17, 18].

The age distribution of participants in our study is comparable to some recent poliomyelitis epidemics. In 2006 in Namibia, all registered cases were more than 14 years old with a male/female ratio of 8.5 [19]. In Albania during a polio outbreak in 1996, 70% of cases were aged between 10 and 34 years and the smallest incidence was registered among the group aged less than 10 years [20–22]. While children are still at highest risk in many settings, as polio eradication efforts progress, more cases may continue to be seen in under-vaccinated young adults, who are prone to particularly severe disease.

### Limitations

The results presented here are derived from routine monitoring data, and as no control group exists, it is not possible to infer causality. In fact it would be unethical to withhold physiotherapy given its established benefits for adults and children with neurological conditions. However future studies could evaluate patients who did not have the benefit of physiotherapy as control subjects.

A high turnover in the physiotherapy team could have led to measurement bias, and also contributed to the missing data seen. In the context of a large epidemic in a single city, the relatively low number of laboratory-confirmed cases is not surprising, though it is possible that some clinically-confirmed cases may have been misclassified. It should also be noted that the duration of follow-up was relatively short. The lasting effects of these physiotherapy interventions were not measured, therefore long-term outcomes are unknown.

## Conclusions

This retrospective analysis describes a small group of patients; formal studies with long-term structured follow-up would be an important next step. Nevertheless these results highlight a potential positive impact of early physiotherapy on muscle strength in patients with poliomyelitis. This may be particularly important given the changing epidemiology of polio as eradication efforts continue. Further research on the possible role of physiotherapy in acute poliomyelitis should be encouraged.

## Abbreviations

AFP: Acute flaccid paralysis
NPEC: National Polio Expert Committee
MSF: Médecins Sans Frontières
IQR: Interquartile Range
WHO: World Health Organization.
